# Sex stratification of the trends and risk of mortality among individuals living with HIV under different transmission categories

**DOI:** 10.1038/s41598-022-13294-y

**Published:** 2022-06-03

**Authors:** Chun-Yuan Lee, Yi-Pei Lin, Hung-Pin Tu, Sheng-Fan Wang, Po-Liang Lu

**Affiliations:** 1 Graduate Institute of Medicine, Kaohsiung Medical University, Kaohsiung, Taiwan (R.O.C.); 2 School of Medicine, College of Medicine, Kaohsiung Medical University, Kaohsiung, Taiwan (R.O.C.); 3 Division of Infectious Diseases, Department of Internal Medicine, Kaohsiung Medical University Hospital, Kaohsiung Medical University, No. 100, Tzyou 1st Road, Kaohsiung, 807 Taiwan (R.O.C.); 4 M.Sc. Program in Tropical Medicine, College of Medicine, Kaohsiung Medical University, Kaohsiung, Taiwan (R.O.C.); 5 Department of Public Health and Environmental Medicine, School of Medicine, College of Medicine, Kaohsiung Medical University, Kaohsiung, Taiwan (R.O.C.); 6 Center for Tropical Medicine and Infectious Disease, Kaohsiung Medical University, Kaohsiung, Taiwan (R.O.C.); 7 Department of Medical Laboratory Science and Biotechnology, Kaohsiung Medical University, Kaohsiung, Taiwan (R.O.C.); 8 Department of Medical Research, Kaohsiung Medical University Hospital, Kaohsiung Medical University, Kaohsiung, Taiwan (R.O.C.); 9 School of Post-Baccalaureate Medicine, College of Medicine, Kaohsiung Medical University, Kaohsiung, Taiwan (R.O.C.); 10 Center for Liquid Biopsy and Cohort Research, Kaohsiung Medical University, Kaohsiung, Taiwan (R.O.C.)

**Keywords:** Infectious diseases, Epidemiology

## Abstract

We retrospectively examined 33,142 persons living with HIV (PLWH) in Taiwan from a nationwide database to assess sex-stratified trends and risk of all-cause mortality under different transmission categories from 1984 to 2016. Overall, 61.25% were men who have sex with men (MSM), 14.37% were men who have sex with women (MSW), 18.32% were male persons who inject drugs (M-PWID), 3.30% were women who have sex with men (WSM), and 2.74% were female PWID (F-PWID). All-cause mortality (per 100 person-years) among heterosexual people and PWID was higher in men (4.04 and 3.39, respectively) than in women (2.93 and 2.18, respectively). In each sex-stratified transmission category, the all-cause mortality reduced substantially from 1984–1996 to 2012–2016, but evolved distinctly from 2007–2011 to 2012–2016. Since 2007–2011, the decline in all-cause mortality has slowed notably in the groups with sexually transmitted HIV, but has increased in PWID, surpassing even that among groups with sexually transmitted HIV in 2012–2016. PLWH with sexually transmitted HIV had lower risks of all-cause mortality than PWID, regardless of sex. Sex and transmission category did not interact significantly on all-cause mortality. Understanding the reasons for the distinct evolving trends of all-cause mortality in each transmission category serves as a reference for developing strategies to reduce mortality in PLWH in Taiwan further.

## Introduction

With the expansion of potent antiretroviral therapy (ART) and a scale-up of the HIV care continuum, mortality has markedly declined among persons living with HIV (PLWH) since the 1980s^1,2^. However, it remains higher than that in the general population^3^. To achieve the goal of ending the HIV/AIDS epidemic by 2030, the Joint United Nations Programme on HIV/AIDS (UNAIDS) has set a target of reducing the number of HIV cases and AIDS-related deaths between 2010 and 2030 by 90% for every country through a further rapid scale-up of HIV testing, prevention, and treatment programs^4,5^. Critical to attaining this target is understanding mortality trends in each country and relevant etiologies, both overall and in specific transmission categories.

Between the 1980s and the mid-2000s, the mortality rate among PLWH in Taiwan declined substantially following a series of mandatory HIV screenings in specific populations (blood donors in 1988, military recruits in 1989, the incarcerated population in 1990, and foreign laborers in 1991). The other factors influencing this reduction in mortality comprised improvements in therapies for opportunistic illnesses, the introduction of universal access to free ART in 1997, and the nationwide initiation of a program offering free anonymous counseling and HIV testing services to sexually active populations in 1997^6,7^. Between the mid-2000s and the 2010s, another series of policies was implemented: an HIV case management program in 2007, the initiation of ART at higher CD4^+^-count thresholds in 2013, and the introduction of a single-tablet regimen in 2016. However, no studies have employed nationwide surveillance data in examining the mortality trends among PLWH in Taiwan since the mid-2000s.

Differences in mortality between different transmission categories (i.e., men who have sex with men [MSM], persons who inject drugs [PWID], and heterosexual people) among PLWH are a subject of debate and concern^8–11^. Depending on the transmission category, PLWH have specific lifestyle or sociodemographic characteristics that are significantly associated with their mortality risk^8–11^. Compared with individuals infected through sexual contact, those infected through intravenous drug use have a significantly higher risk of all-cause mortality^6,8,11,12^. This includes mortality attributable to external causes, particularly overdose and suicide^9,10,13^, as well as virological or immunological failure^12^ or non-AIDS infectious diseases^14^. Assessments of differences in mortality between MSM and men who have sex with women (MSW) have yielded conflicting results. Some reports have indicated that MSM have higher survival rates than MSW due to better adherence to ART^6,15–17^, whereas two retrospective cohort studies, conducted in Brazil^18^ and Denmark^19^, respectively, did not support a difference in mortality between MSM and MSW.

Notably, differences in mortality between different transmission categories may be confounded or modified by sex. Studies conducted in Europe^20^, China^15,21^, Kenya^22^, and South Africa^23^ have reported that female PLWH have a lower risk of mortality than male PLWH. This is possibly because of women’s better adherence to ART^15,21^, lower rate of substance use^24^, or more favorable immune response to combined ART (cART)^25^. Therefore, a lower proportion of HIV acquisition through injecting drugs in women may explain potential differences in mortality between transmission categories^26,27^. However, no sex differences in all-cause mortality were observed in the United States or Canada^20^. Moreover, differences in mortality by transmission category may also be modified by sex differences. A study determined that compared with male PWID, female PWID were more likely to have high-risk sex^28^, thereby increasing their risk of mortality from sexually transmitted diseases such as hepatitis. A cohort study conducted in Europe found greater sex differences in all-cause mortality among heterosexual people than among PWID (interaction *P* = 0.043)^20^. However, a retrospective cohort study conducted in Puerto Rico in 2003–2014 revealed that the relative risk of mortality between PWID and heterosexual people was comparable among the sexes^29^. Given that the sex distribution of heterosexual people and PWID can differ considerably^26,27^, sex-stratified analyses are warranted to quantify mortality among PLWH under distinct transmission categories, as well as to address persistent disparities or health inequities more systematically.

In this retrospective nationwide cohort study, we first explored trends in overall and sex-stratified all-cause mortality among PLWH in Taiwan under various transmission categories between 1984 and 2016. We then estimated the differences in overall and sex-stratified all-cause mortality between different transmission categories.

## Results

### Patient enrollment

**Figure 1:**
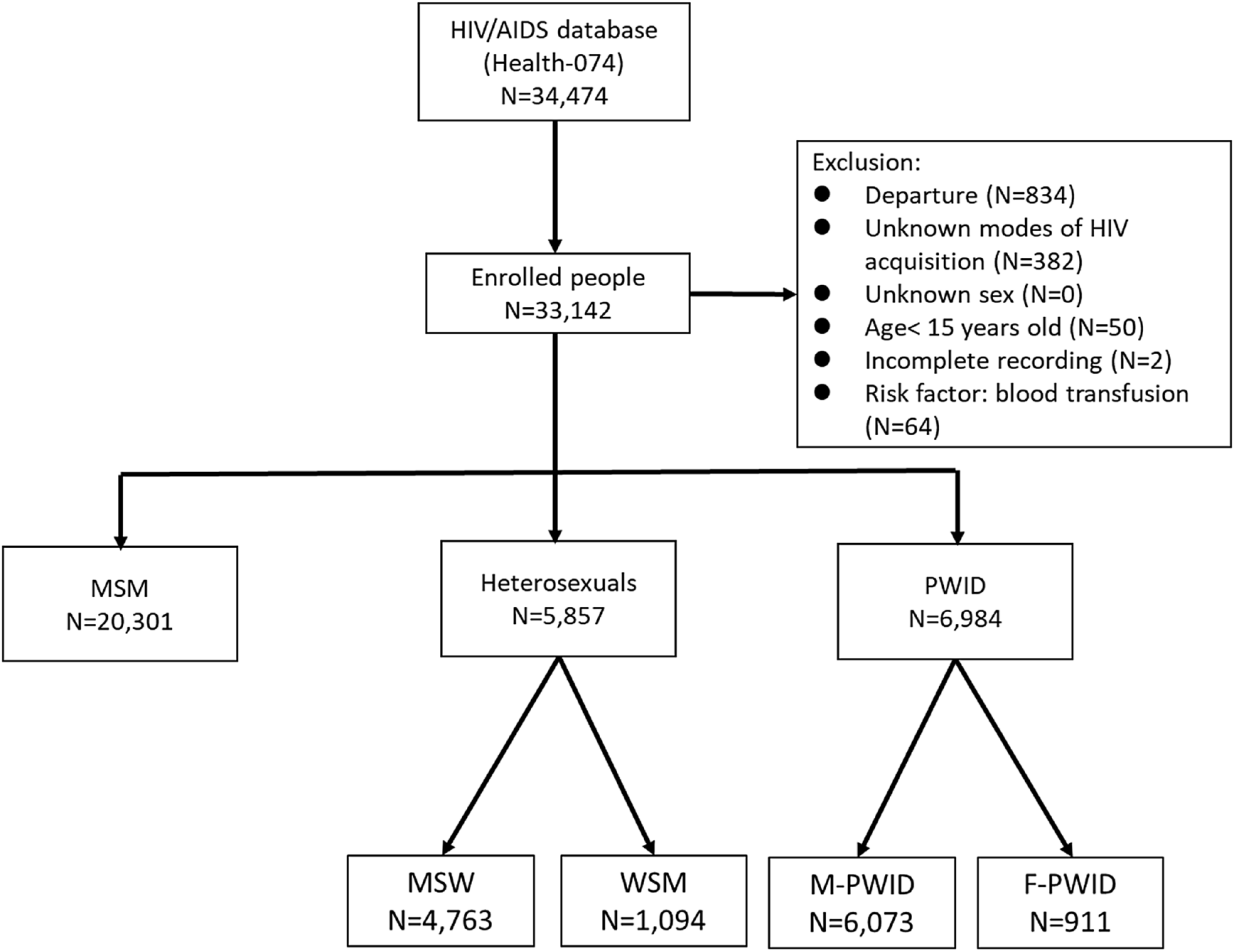
Study flowchart. Abbreviations: AIDS, acquired immunodeficiency syndrome; F-PWID, female persons who inject drugs; HIV, human immunodeficiency virus; PWID, persons who inject drugs; M-PWID, male persons who inject drugs; MSM, men who have sex with men; MSW, men who have sex with women; WSM, women who have sex with men.

After the exclusion of 1332 individuals (departures, n = 834; unknown transmission categories, n = 382; aged < 15 years, n = 50; incomplete records, n = 2; HIV acquisition through blood transfusions, n = 64), 33,142 people were enrolled: 20,301 MSM; 5857 heterosexual people (comprising 4763 MSW and 1094 WSM); and 6984 PWID (comprising 6073 male PWID [M-PWID] and 911 female PWID [F-PWID]; Fig. 1).

### Baseline sociodemographic characteristics among various transmission categories, overall and by sex

The baseline sociodemographic characteristics of the patient groups are summarized in Table 1. Almost half of the individuals were aged ≤ 30 years (48.145%). Furthermore, most people were male (93.95%), MSM (61.25%), unmarried (78.57%), employed (60.12%), and had been diagnosed at designated HIV hospitals (62.71%). Moreover, 35.42% were diagnosed in 1997–2006, and 39.86% were diagnosed in Taipei.

**Table 1:** Comparison of sociodemographic characteristics of enrolled people across key populations, overall and within each sex.

	Total n = 33,142	Overall				Male				Female		
		MSM N = 20,301	HEX N = 5857	PWID N = 6984	*P*-value	MSM N = 20,301	MSW N = 4763	M-PWID N = 6073	*P*-value	WSM N = 1094	F-PWID N = 911	*P*-value
Age group at HIV presentation, n (%)					< 0.001				< 0.001			< 0.001
≤ 30	15,954 (48.14)	11,895 (58.59)	1655 (28.26)	2404 (34.42)		11,895 (58.59)	1335 (28.03)	1918 (31.58)		320 (29.25)	486 (53.35)	
> 30– ≤ 50	15,036 (45.37)	7869 (38.76)	2929 (50.01)	4238 (60.68)		7869 (38.76)	2405 (50.49)	3828 (63.03)		524 (47.9)	410 (45.01)	
> 50	2152 (6.49)	537 (2.65)	1273 (21.73)	342 (4.90)		537 (2.65)	1023 (21.48)	327 (5.38)		250 (22.85)	15 (1.65)	
Male sex, n (%)	31,137 (93.95)	20,301 (100.00)	4763 (81.32)	6073 (86.96)	< 0.001							
Period of HIV diagnosis, n (%)					< 0.001				< 0.001			< 0.001
1984–1996	1046 (3.16)	533 (2.63)	466 (7.96)	47 (0.67)		533 (2.63)	387 (8.13)	44 (0.72)		79 (7.22)	3 (0.33)	
1997–2006	11,739 (35.42)	4256 (20.96)	2440 (41.66)	5043 (72.21)		4256 (20.96)	1996 (41.91)	4397 (72.4)		444 (40.59)	646 (70.91)	
2007–2011	9026 (27.23)	5893 (29.03)	1576 (26.91)	1557 (22.29)		5893 (29.03)	1299 (27.27)	1346 (22.16)		277 (25.32)	211 (23.16)	
2012–2016	11,331 (34.19)	9619 (47.38)	1375 (23.48)	337 (4.83)		9619 (47.38)	1081 (22.7)	286 (4.71)		294 (26.87)	51 (5.6)	
Marriage, n (%)					< 0.001				< 0.001			< 0.001
Unknown	82 (0.25)	53 (0.26)	12 (0.2)	17 (0.24)		53 (0.26)	9 (0.19)	15 (0.25)		3 (0.27)	2 (0.22)	
No	26,040 (78.57)	19,304 (95.09)	2825 (48.23)	3911 (56.00)		19,304 (95.09)	2593 (54.44)	3610 (59.44)		232 (21.21)	301 (33.04)	
Yes	7020 (21.18)	944 (4.65)	3020 (51.56)	3056 (43.76)		944 (4.65)	2161 (45.37)	2448 (40.31)		859 (78.52)	608 (66.74)	
Occupation, n (%)					< 0.001				< 0.001			< 0.001
Unknown	1690 (5.1)	1284 (6.32)	288 (4.92)	118 (1.69)		1284 (6.32)	232 (4.87)	104 (1.71)		56 (5.12)	14 (1.54)	
Student	2740 (8.27)	2534 (12.48)	198 (3.38)	8 (0.11)		2534 (12.48)	182 (3.82)	6 (0.10)		16 (1.46)	2 (0.22)	
Unemployment	8786 (26.51)	2908 (14.32)	1655 (28.26)	4223 (60.47)		2908 (14.32)	1129 (23.70)	3592 (59.15)		526 (48.08)	631 (69.26)	
Employment	19,926 (60.12)	13,575 (66.87)	3716 (63.45)	2635 (37.73)		13,575 (66.87)	3220 (67.60)	2371 (39.04)		496 (45.34)	264 (28.98)	
Specimen source					< 0.001				< 0.001			< 0.001
Designated HIV hospital	20,785 (62.71)	15,882 (78.23)	3932 (67.13)	971 (13.9)		15,882 (78.23)	3245 (68.13)	819 (13.49)		687 (62.80)	152 (16.68)	
Military screening	965 (2.91)	839 (4.13)	108 (1.84)	18 (0.26)		839 (4.13)	107 (2.25)	18 (0.30)		1 (0.09)	0 (0.00)	
Blood donation	1325 (4.00)	828 (4.08)	469 (8.01)	28 (0.4)		828 (4.08)	405 (8.50)	24 (0.40)		64 (5.85)	4 (0.44)	
Imprisonment screening	4743 (14.31)	286 (1.41)	237 (4.05)	4220 (60.42)		286 (1.41)	213 (4.47)	3756 (61.85)		24 (2.19)	464 (50.93)	
Others	5324 (16.06)	2466 (12.15)	1111 (18.97)	1747 (25.01)		2466 (12.15)	793 (16.65)	1456 (23.97)		318 (29.07)	291 (31.94)	
HIV diagnosis region, n (%)					< 0.001				< 0.001			< 0.001
Taipei	13,209 (39.86)	9717 (47.9)	2074 (35.4)	1418 (20.3)		9717 (47.9)	1667 (35.00)	1231 (20.27)		407 (37.20)	187 (20.53)	
Northern Taiwan	4551 (13.73)	2564 (12.63)	786 (13.42)	1201 (17.20)		2564 (12.6)	606 (12.72)	966 (15.91)		180 (16.45)	235 (25.80)	
Central Taiwan	5613 (16.94)	3141 (15.47)	1093 (18.66)	1379 (19.75)		3141 (15.47)	882 (18.52)	1217 (20.04)		211 (19.29)	162 (17.78)	
Southern Taiwan	3403 (10.27)	1441 (7.10)	726 (12.40)	1236 (17.70)		1441 (7.1)	589 (12.37)	1117 (18.39)		137 (12.52)	119 (13.06)	
Kaoping area	5780 (17.44)	3080 (15.17)	1018 (17.38)	1682 (24.08)		3080 (15.17)	884 (18.56)	1482 (24.40)		134 (12.25)	200 (21.95)	
Eastern Taiwan	586 (1.77)	358 (1.76)	160 (2.73)	68 (0.97)		358 (1.8)	135 (2.83)	60 (0.99)		25 (2.29)	8 (0.88)	
AIDS event during observation	15,254 (46.03)	9106 (44.85)	3616 (61.74)	2532 (36.25)	< 0.001	9106 (44.85)	3016 (63.32)	2200 (36.23)	< 0.01	600 (54.84)	332 (36.44)	< 0.01

Abbreviation: AIDS, acquired immunodeficiency syndrome; HEX, heterosexual; HIV, human immunodeficiency virus; PWID, persons who inject drugs; MSM, men who have sex with men; MSW, men who have sex with women; WSM, women who have sex with men.

Among the transmission categories, significant sociodemographic, specimen source, and geographic differences were observed. After stratification by sex, the sociodemographic characteristics and specimen sources of male PLWH exhibited a similar distribution to that of the overall study population.

### Overall all-cause mortality in various transmission categories

Overall, the median follow-up duration was 6.17 years (interquartile range [IQR] = 8). The median follow-up duration for MSM was significantly shorter than that for heterosexual people and PWID (*P* < 0.001, Table 2). The differences among transmission categories observed during follow-up were comparable among sexes.

**Table 2:** Association of transmission categories with all-cause mortality, overall and within each sex.

	Total N = 33,142	Overall				Male				Female		
		MSM N = 20,301	HEX N = 5857	PWID N = 6984	*p*-value	MSM N = 20,301	MSW N = 4763	M-PWID N = 6073	*p*-value	WSM N = 1094	F-PWID N = 911	*P*-value
Total follow-up (person-years)	227,156.79	121,723.78	43,734.75	61,698.26		121,723.78	35,193.36	53,443.72		8541.39	8254.54	
Median follow-up (Years, IQR)	6.17 (8)	4.75 (6.50)	6.58 (9.17)	10.25 (4.17)	< 0.001	4.75 (6.50)	6.50 (9.08)	10.17 (4.33)	< 0.001	6.75 (9.49)	10.33 (3.16)	< 0.001
All-cause deaths, n (%)	5412 (16.33)	1746 (8.6)	1672 (28.55)	1994 (28.55)	< 0.001	1746 (8.60)	1422 (29.86)	1814 (29.87)	< 0.001	250 (22.85)	180 (19.76)	0.09
Mortality, per100 person-years (95%CI)	2.38 (2.32–2.45)	1.43 (1.37–1.50)	3.82 (3.65–4.01)	3.23 (3.10–3.37)	< 0.001	1.43 (1.37–1.50)	4.04 (3.84–4.25)	3.39 (3.24–3.55)	< 0.001	2.93 (2.59–3.31)	2.18 (1.89–2.52)	< 0.001
Crude HR (95% CI)		0.42 (0.39–0.45)	1.16 (1.08–1.23)	Reference		0.40 (0.37–0.43)	1.16 (1.08–1.25)	Reference		1.33 (1.09–1.62)	Reference	
*P*-value		< 0.001	< 0.001			< 0.001	< 0.001			0.004		
Adjusted HR* (95% CI)		0.34 (0.32–0.38)	0.50 (0.45–0.56)	Reference		0.35 (0.32–0.39)	0.51 (0.45–0.57)	Reference		0.45 (0.33–0.62)	Reference	
*P*-value		< 0.001	< 0.001			< 0.001	< 0.001			< 0.001		
*P* for interaction**					0.356							

Abbreviation: AIDS, acquired immunodeficiency syndrome; CI, confidence interval; HEX, heterosexual; HIV, human immunodeficiency virus; PWID, persons who inject drugs; PWID-M, male injection drug use; PWID-W, female injection drug use; IQR, interquartile range; MSM, man who have sex with man; MSW, man who has sex with woman; WSM, woman who has sex with man.

*Adjusted hazard ratio (HR) with 95% CI was estimated after adjustment for age group, sex, period of HIV diagnosis, HIV transmission category, marital status, occupation, specimen source, HIV diagnosis region, and AIDS event in all set; and age group, period of HIV diagnosis, HIV transmission category, marital status, occupation, specimen source, HIV diagnosis region, and AIDS event in sex-stratified analyses.

**We found no significant interaction between sex and HIV-related risk on the all-cause mortality.

Over the study period (1984–2016), 5412 of the 33,142 individuals (16.3%) died (Table 2). The median age at death was 42 (IQR, 35–50) years, increasing from 39 years in 1984–1996 to 44 years in 2012–2016.

Regarding the HIV transmission category, MSM, heterosexual people, and PWID accounted for 32.26% (1746/5412), 30.9% (1672/5412), and 36.84% (1994/5412) of mortality cases, respectively (Table 2). The proportion of all-cause mortality differed significantly among different transmission categories (MSM vs. heterosexual people vs. PWID: 8.6% vs. 28.55% vs. 28.55%, *P* < 0.001). The overall all-cause mortality (per 100 person-years) was 2.38 (95% CI 2.32–2.45), with the highest rate among heterosexual people (3.82 [95% CI 3.65–4.01]) and the lowest rate among MSM (1.43 [95% CI 1.37–1.50]; Table 2).

### All-cause mortality in various transmission categories by sex

Under sex stratification, the proportions of all-cause mortality differed significantly among transmission categories (in men, MSM vs. heterosexual men vs. male PWID: 8.6% vs. 29.86% vs. 29.87%, *P* < 0.001; in women, heterosexual women vs. female PWID: 22.85% vs. 19.76%, *P* < 0.001). All-cause mortality among heterosexual people and PWID was higher in men (4.04 [95% CI 3.84–4.25] and 3.39 [3.24–3.55], respectively) than in women (2.93 [95% CI 2.59–3.31] and 2.18 [1.89–2.52], respectively).

### Evolving trends of all-cause mortality in various transmission categories across the four study periods

**Figure 2:**
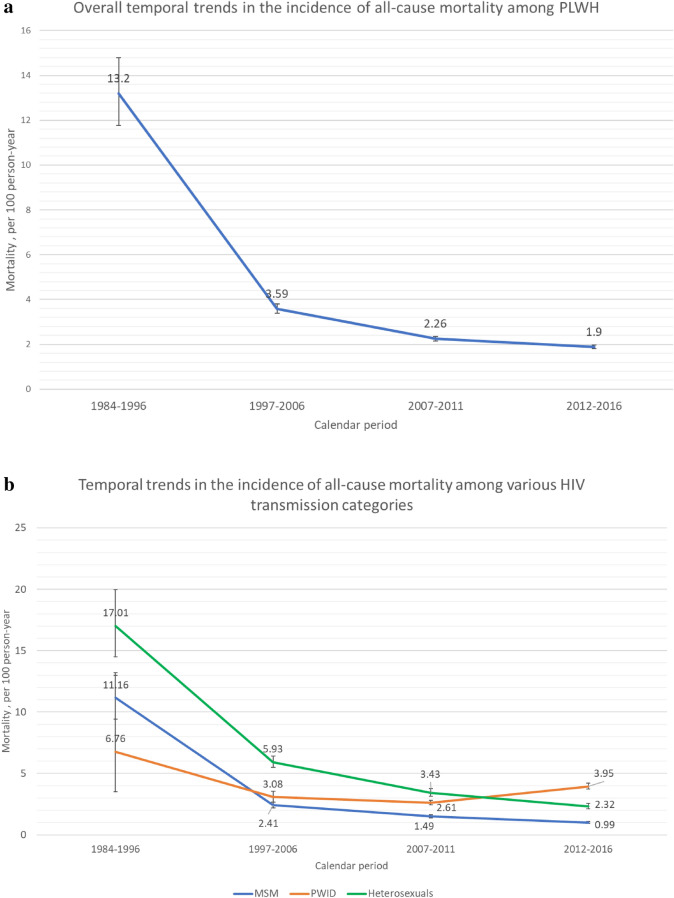
Temporal trends in the incidence of mortality (100 person-years) among (**a**) all PLWH and (**b**) various transmission categories from 1984 to 2016. Abbreviations: HIV, human immunodeficiency virus; PWID, persons who inject drugs; MSM, men who have sex with men; PLWH, persons living with HIV.

Across the four periods of HIV diagnosis, the all-cause mortality rate (per 100 person-years) decreased substantially from 13.20 (11.76–14.80) in 1984–1996 to 3.59 (3.40–3.80) in 1997–2006 before declining slowly to 1.90 (1.82–1.98) in 2012–2016, corresponding to a 85.6% reduction overall (Fig. 2a). Between 2007–2011 and 2012–2016, all-cause mortality decreased by 36%.

In the transmission categories, trends in all-cause mortality evolved distinctly across the study periods. Overall, the mortality rate (per 100 person-years) was the highest in 1984–1996 for groups infected through sexual contact (11.16 [95% CI 9.43–13.21] in MSM and 17.00 [95% CI 14.48–19.98] in heterosexual people). It then declined substantially in 2012–2016 (0.99 [95% CI 0.92–1.07] in MSM and 2.32 [95% CI 2.10–2.54] in heterosexual people, corresponding to 91.1% and 86.4% reductions in all-cause mortality, respectively). Among PWID, the all-cause mortality rate (per 100 person-years) peaked in 1984–1996 (6.76 [95% CI 3.52–12.99]) before declining in 2007–2011 (2.61 [95% CI 2.43–2.81]) and then increasing in 2012–2016 (3.95 [95% CI 3.72–4.20]). The overall reduction in all-cause mortality was 41.6%. Between 2007–2011 and 2012–2016, mortality decreased by 33.6% among MSM and by 32.7% among heterosexual people but increased by 51.3% among PWID. The highest mortality rate in 2012 − 2016 was in the PWID group (Fig. 2b).

Early mortality (i.e., mortality within 180 days of HIV diagnosis) among MSM was 42.22% in 1984–1996 and 27.72% in 2012–2016 (34.34% reduction). For the same periods, the corresponding rates among heterosexual people were 45.95% and 21.48%, respectively (53.25% reduction). As for PWID, the corresponding rates were 11.1% and 1.42%, respectively (87.2% reduction).

### Evolving trends of all-cause mortality in various transmission categories across the four study periods by sex

In both sexes, the trend of all-cause mortality in different at-risk subgroups was similar to that for the overall cohort; however, mortality incidence was lower in women than in men.

**Figure 3:**
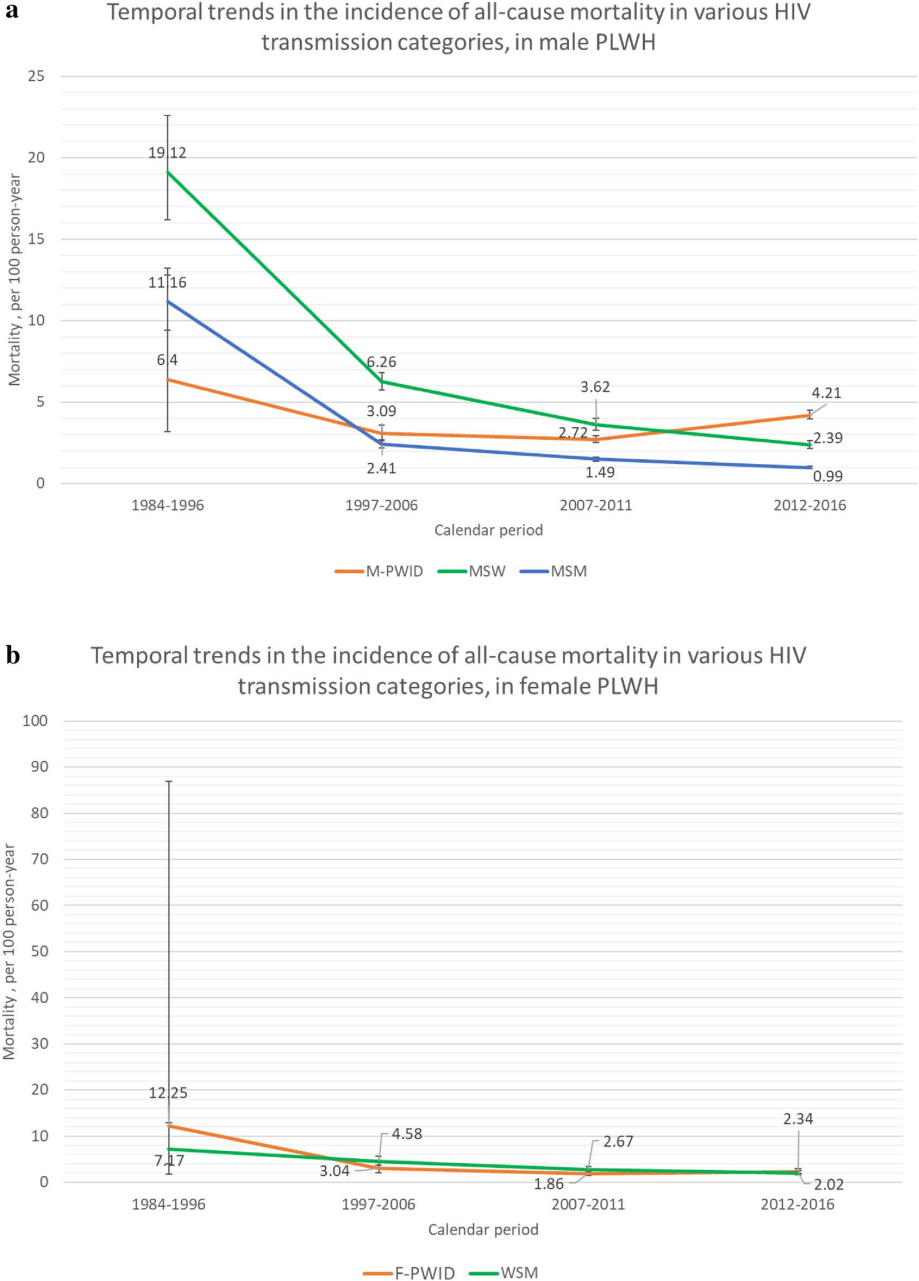
Temporal trends in the incidence of mortality (100 person-years) in various HIV transmission categories from 1984 to 2016 in (**a**) male PLWH and (**b**) female PLWH. Abbreviations: F-PWID, female persons who inject drugs; HIV, human immunodeficiency virus; PWID, persons who inject drugs; M-PWID, male persons who inject drugs; MSM, men who have sex with men; MSW, men who have sex with women; PLWH, persons living with HIV; WSM, women who have sex with men.

In men, the all-cause mortality (per 100 person-years) was the highest in 1984–1996 for groups infected through sexual contact (11.16 [95% CI 9.43–13.21] in MSM and 19.12 [95% CI 16.17–22.61] in MSW). In 2012–2016, this rate declined considerably (0.99 [95% CI 0.92–1.07] in MSM and 2.39 [95% CI 2.54–2.10] in MSW, corresponding to 91.1% and 87.5% reductions, respectively). For M-PWID, the mortality rate (per 100 person-years) peaked in 1984–1996 (6.40 [95% CI 3.20–12.80]), dropped in 2007–2011 (2.72 [95% CI 2.52–2.94]), and increased slightly in 2012–2016 (4.21 [95% CI 3.96–4.49]), with an overall 34.2% reduction (Fig. 3a). From 2007–2011 to 2012–2016, mortality among MSM and MSW decreased by 33.6% and 34.0%, respectively, and increased by 54.8% for M-PWID.

In female PLWH, mortality (per 100 person-years) was the highest in 1984–1996 among both WSM (7.17 [95% CI 3.97–12.94]) and F-PWID (12.25 [95% CI 1.73–86.93]). Among WSM, all-cause mortality declined by 71.8% to 2.02 (95% CI 1.61–2.54) in 2012–2016. For F-PWID, all-cause mortality declined in 2007–2011 (1.86 [95% CI 1.47–2.35]) and then increased slightly in 2012–2016 (2.34 [95% CI 1.90–2.89]; 80.9% overall reduction; Fig. 3b). From 2007–2011 to 2012–2016, mortality decreased by 24.3% among WSM and increased by 25.8% among F-PWID.

### Key population-level variations in all-cause mortality

Among all PLWH, with PWID as the reference category, the crude hazard ratio (cHR) for all-cause mortality was significantly reduced for MSM (cHR: 0.42, 95% CI 0.39–0.45, *P* < 0.001). The same was true for the adjusted HR (aHR; 0.34, 95% CI 0.32–0.38, *P* < 0.001). The cHR of all-cause mortality for heterosexual people, with PWID as the reference category, was 1.16 (95% CI 1.08–1.25, *P* < 0.001). This result was inverted (aHR: 0.50, 95% CI 0.45–0.56, *P* < 0.001) after adjustment in the multivariable Cox regression model (Table 2).

In multivariable Cox regression models, male sex (aHR = 1.39; 95% CI 1.24 − 1.56) was associated with an elevated risk of all-cause mortality. No significant transmission category–sex interaction was noted (*P* = 0.356).

### Key population-level variations in all-cause mortality by sex

Under sex stratification, in male PLWH (with PWID as the reference category), the cHR of all-cause mortality among MSM (cHR: 0.40, 95% CI 0.37–0.43, *P* < 0.001) did not change significantly following Cox regression (aHR: 0.35, 95% CI 0.32–0.39, *P* < 0.001). The cHR of all-cause mortality among heterosexual men (cHR: 1.16, 95% CI 1.08–1.25, *P* < 0.001) was inverted following Cox regression (aHR: 0.51, 95% CI 0.45–0.57, *P* < 0.001).

In female PLWH (with PWID as the reference category), the cHR of all-cause mortality for heterosexual women (cHR: 1.33, 95% CI 1.09–1.62, *P* = 0.004) was inverted following Cox regression (aHR: 0.45, 95% CI 0.33–0.62, *P* < 0.001).

## Discussion

Since the 1990s, Taiwan has implemented measures to enable the early diagnosis of HIV through active and passive surveillance systems^30^. Under improvements in therapies for opportunistic illnesses and the introduction of universal access to free ART, substantial declines in all-cause mortality from the 1980s to mid-2000s have been observed. Starting in the mid-2000s, this decline slowed gradually. The curve of decline is consistent with those presented in studies conducted in other developed regions^31–33^. However, the all-cause mortality rate has evolved distinctly in different sex-stratified transmission categories since the mid-2000s. Specifically, the decline in all-cause mortality has slowed notably in groups with sexually transmitted HIV (MSM, MSW, WSM) since 2007–2011. All-cause mortality among PWID has increased since 2007–2011, even surpassing all-cause mortality among groups with sexually transmitted HIV in 2012–2016. Therefore, a further exploration of the possible reasons of distinct evolution of all-cause mortality between 2007–2011 and 2012–2016 in each sex-stratified transmission category would be conducive to inform relevant policies, and then to further reduce mortality in PLWH in Taiwan.

First, the slowing of the decline in all-cause mortality among MSM, MSW, WSM in our study population may be partly explained by the persistently high prevalence of late HIV presentation among those with sexually transmitted HIV in Taiwan over the past 2 decades^34,35^. This high prevalence, which contributes to AIDS-related deaths^30,36^, may partially nullify the benefits conferred by the reduction of mortality from improved medical care, the implementation of universal ART, and the introduction of a single-tablet regimen. Second, 2003 notably marked the beginning of the rapid spread of the circulating recombinant form (CRF) 07_BC HIV strain among PWID^37,38^. In 2005, the implementation of harm reduction therapy rapidly contained the epidemic^39^, and the annual incidence of HIV cases among PWID has remained low since. The rising mortality rate among PWID in 2012–2016 in the present study may be due to a cohort effect stemming from the CRF07_BC epidemic in 2003. Considering the slow immunological progression caused by HIV CRF07_BC strain^40^, premature aging among PLWH^41^, and higher exposure to harmful lifestyles among PWID^9,10,13,41^, non-AIDS-related deaths are postulated to be the primary category of rising mortality observed in 2012–2016. In summary, to further reduce mortality in PLWH in Taiwan, strategies should be tailored to specific transmission categories: optimizing measures to expand targeted HIV testing for sexually at-risk populations to enhance early diagnosis of HIV infection, and intensifying surveillance of non-AIDS comorbidities and interventions to reduce harmful lifestyles among PWID PLWH.

Because of the limited availability of data on the causes of mortality, we could not evaluate the evolving trends of AIDS-related and non-AIDS-related deaths among PLWH. According to other studies, the substantial reduction in overall mortality over the past 3 decades has mainly been driven by AIDS-related causes in countries where sexual contact is the predominant route of HIV transmission^32,42,43^. Because early mortality is a proxy for AIDS-related mortality^30^, the significant decline in the proportion of early mortality across the three transmission categories suggests a significant trend of decline in AIDS deaths across these transmission categories. However, in 2012–2016, the early mortality rate in the groups with sexually transmitted HIV was not particularly low at 20%. Across the four periods of HIV diagnosis, deaths occurring > 180 days following HIV diagnosis remained considerable among PWID. This indicates the persistently high burden of non-AIDS-related deaths among PWID. Further examination of cause-specific mortality is necessary to elucidate its trends in various transmission categories and then guide the implementation of relevant interventions.

In the present study, the absolute all-cause mortality was higher among heterosexual people than among PWID (3.82 vs. 3.23 per 100 person-years). However, in both sexes, the association of all-cause mortality between PWID and heterosexual people was inverted after adjustment for confounders. Among PLWH, compared with PWID, heterosexual people are more prone to experiencing an AIDS event at presentation and during follow-up^26,35^. Furthermore, they tend to be older than PWID at the time of HIV diagnosis^26^. Both an AIDS event and older age at HIV diagnosis can increase the risk of all-cause mortality^30^. We determined that the aHR for the effect of heterosexuality (vs. PWID) on all-cause mortality in a model excluding age groups and AIDS events (supplementary file 1) was comparable to the crude HR for the effect of heterosexuality (vs. PWID) on all-cause mortality in the entire cohort (Table 2). When either age group or AIDS events were considered in the model, the aHR was reduced by more than 10%, suggesting that these two variables are confounders in the association between heterosexuality (vs. PWID) and all-cause mortality (Supplementary Table S1 online).

Consistent with findings presented in other studies, PWID had the highest risk of all-cause mortality in our study population^6,8,11,12^. The causes of this increased risk of all-cause mortality among PWID may be external, such as overdose, suicide^9,10,13^, or repeated intravenous injections, which can lead to non-AIDS infectious diseases (e.g., hepatitis C virus coinfection and infective endocarditis)^14,42,44^. Moreover, PWID frequently face numerous socioeconomic challenges, such as low education levels, incarceration (previous or current)^45^, economic difficulties, lack of social and family support, and associated psychiatric comorbidities^46,47^. These difficulties may prevent the successful and continual engagement and retention of PWID on the HIV care continuum.

The evidence on whether all-cause mortality differs between MSM and MSW is conflicting. Nationwide cohort studies conducted in Denmark and Brazil have revealed no such differences^18,19^. By contrast, in line with the results of a study conducted in China, we observed higher survival among MSM than among MSW^48^. A possible explanation is that MSM have closer connections to the HIV care continuum and better compliance with cART than do MSW. A UK investigation reported that MSM had a lower risk of virological failure than heterosexual people^16^. Furthermore, a study examining data from Thailand, Hong Kong, Malaysia, the Philippines, and Indonesia indicated that ART adherence was higher among MSM than among heterosexual people^17^.

Studies exploring sex differences in mortality have presented contradictory findings. A surveillance study of 19 PLWH cohorts in Europe and North America reported lower rates of all-cause mortality among female PLWH in Europe but no sex differences in Canada or the United States^20^. A retrospective cohort study conducted in China determined that male PLWH had higher risks of all-cause mortality than female PLWH^15^. These discrepancies may reflect differences in socioeconomic profiles or unmeasured confounders that correspond to uneven risks of unhealthy lifestyle (e.g., smoking or other lifestyle factors) among men and women^49^. Consistent with our findings, a global systematic review and meta-analysis of 65 studies concluded that female PLWH had higher survival than male PLWH in both resource-rich and resource-limited regions^50^. Although the studies related to the role of sex on adherence to ART are inconsistent^15,21,51^, the higher risk of all-cause mortality in male PLWH may be due to their higher risk of progression to AIDS, immunological and virological failure, and lower adherence to ART and care^15,21^. However, sex differences in immune responses may also complicate the association between non-AIDS-related mortality and sex. Female PLWH tend to have higher levels of immune activation and interferon-stimulated gene expression than male PLWH^52,53^, which likely influence the development of sex-related non-AIDS comorbidities. Moreover, male PLWH have higher risks of dying from non-AIDS-related causes, including substance use and external factors^20^. Therefore, future studies should elucidate the sex differences in AIDS-related deaths and non-AIDS-related deaths among PLWH in Taiwan. A European study found greater sex differences in all-cause mortality among heterosexual people than among PWID^20^. In the present study, we didn’t observe significant transmission category–sex interaction. This between-study discrepancy might be due to the uneven distribution of risks of sex-related non-AIDS comorbidities between PWID and heterosexual people in European populations rather in our study population.

This study has two key strengths. First, it is the first study to conduct sex-stratified analyses of all-cause mortality among PLWH in different transmission categories in Taiwan for the past 3 decades. Second, the nationwide and population-based design, combined with the extended follow-up duration and almost complete follow-up records, minimized selection and referral biases. However, our study has several limitations. First, HIV acquisition was self-reported and categorized into mutually exclusive classifications. Therefore, we could not identify people in more than one type of HIV exposure group (e.g., MSM who may also be PWID), which would result in higher risks of all-cause mortality^6^. Second, although we divided the events of all-cause mortality by time since HIV diagnosis to provide a proxy for AIDS-related mortality (if the event developed within 180 days of diagnosis), we were unable to precisely differentiate between AIDS-related and non-AIDS-related deaths because of the limited information available on specific causes of death^18^. Third, we lacked information on such well-established confounders as social trust^54^, income, education level, substance use^24^ and alcohol^55^ use, comorbidities, timing of ART initiation and classes of antiretroviral agents, and adherence to HIV care and ART. These unexamined variables may account for some of the observed associations of all-cause mortality between different transmission categories. Finally, health-care providers in Taiwan are not required to record patients’ gender identity on reporting patient information. However, gender identity may be associated with HIV risk behaviors and socioeconomic status, which may in turn affect compliance with ART, HIV care, and mortality^56,57^.

In summary, we determined that under the implementation of a series of HIV prevention and management strategies, the trend of all-cause mortality among PLWH in Taiwan declined from 1984 to 2016. This decline leveled off after 2007, and the evolution of the trend in each transmission category became distinct. These observations indicate the emerging need to analyze specific causes of death among PLWH and to implement interventions relevant to individuals in each transmission category. The elevated risk of all-cause mortality among heterosexual people was inverted following adjustment for the confounders of AIDS events and older age at diagnosis. In both sexes, differences in all-cause mortality associated with the transmission categories remained, with PWID being the most at-risk population, followed by heterosexual people and MSM. Our data extend the understanding of all-cause mortality in PLWH in Taiwan and serve as a reference for the development of intervention strategies for each mode of HIV acquisition.

## Methods

### Data sources, study design, and setting

We retrospectively screened for new diagnoses of PLWH from a nationwide HIV/AIDS database under the Taiwan Centers for Disease Control (TCDC)-operated Notifiable Diseases Surveillance System (NDSS). The researchers can apply to the Health and Welfare Data Science Center, Ministry of Health and Welfare for the HIV/AIDS database. The HIV/AIDS database updated annually^26,35^. Health-care providers must report patient information to TCDC-operated NDSS—including the national identification number, sex, date of birth, date of HIV/AIDS diagnosis, occupation, specimen source, HIV transmission categories, region of HIV diagnosis, and marital status—within 24 h of HIV diagnosis. In this context, AIDS reporting requirements are based on the case definition of AIDS presented by the US Centers for Disease Control and Prevention in 1993^58^. The HIV/AIDS database records an AIDS event only once per patient. Trained public health personnel maintain records on the status of each individual—that is, whether they are alive, dead, or no longer in Taiwan (designated as departure).

All data in the HIV/AIDS database are deidentified to protect personal privacy, and researchers analyze the data anonymously under the ambit of personal data protection laws. The Institutional Review Board of Kaohsiung Medical University Hospital approved the study protocol [approval no. KMUHIRB-E (II)-20200084]. The informed consent was waived by the Institutional Review Board of Kaohsiung Medical University Hospital. The study was conducted in adherence to the principles of the Declaration of Helsinki, and the use of the TCDC database for the present investigation was authorized by Taiwan’s Ministry of Health and Welfare, with all authors signing a confidentiality agreement.

### Study population

Details of the enrollment process were described previously^26^. In brief, we searched the database for data on PLWH with new HIV diagnoses in the period from January 1984 to December 2016. PLWH who were aged < 15 years, had a departure status, unknown HIV transmission route, unknown sex, or incomplete records were excluded from further analysis, as were individuals belonging to a transmission category irrelevant to our study (e.g., blood transfusion). We examined data from the date of HIV diagnosis to each individual’s death or December 31, 2016, whichever occurred first. The enrolled individuals were first grouped by transmission category (MSM, heterosexual people, and PWID) and then categorized into subgroups by sex: MSM, MSW, WSM, M-PWID, and F-PWID.

### Working definitions

We analyzed covariates measured at baseline. These are listed as follows: age (≤ 30, 31–50, and ≥ 51 years), sex, period of HIV diagnosis (1984–1996, before highly active ART [HAART]; 1997–2006, early-HAART; 2007–2011, mid-HAART; or 2012–2016, contemporary HAART), transmission category, marital status (unknown, unmarried, or married), occupation (unknown, student, unemployed, or employed), specimen source (designated hospital, military screening, blood donation, imprisonment screening, or others), and region of HIV diagnosis^35^ (Taipei, northern Taiwan, central Taiwan, southern Taiwan, the Kaoping area, and eastern Taiwan).

The time points of the mortality events were categorized according to the timing of their occurrence after HIV diagnosis (i.e., ≤ 180 and > 180 days) as a proxy for possible AIDS-related mortality^30^. Because of the limited availability of data on the causes of mortality in the reporting system, the reported deaths were considered under the umbrella of all-cause mortality.

### Statistical analysis

To assess the distribution of sociodemographic variables, specimen source, AIDS events during observation, and all-cause mortality rates per 100 person-years during observation (overall, by sex, and by transmission category), we applied the Kruskal–Wallis and chi-square tests to continuous and categorical variables, respectively.

In comparisons of all-cause mortality (overall and sex stratified) among MSM or heterosexual people with PWID, we employed Cox proportional hazards models in assessing hazard ratios (HRs; both unadjusted and adjusted) with 95% CIs. Potential risk factors—namely age group, sex, period of HIV diagnosis, at-risk population, marriage, occupation, specimen source, and region of HIV diagnosis—were incorporated into the Cox proportional hazards model, with AIDS events as a time-updated variable.

The cHR of all-cause mortality in a comparison of heterosexual people and PWID was inverted following adjustment for confounders in the multivariable Cox regression model. To investigate these potential confounders, we employed various models to estimate the aHR of all-cause mortality in a comparison of heterosexual people and PWID.

After excluding MSM, we tested for interactions between transmission category and sex in separate Cox proportional hazards models. Scaled Schoenfeld residuals and Schoenfeld-like residuals were used to test the proportional hazards assumption by age group, sex, period of HIV diagnosis, region of HIV diagnosis, at-risk population, marital status, occupation, and specimen source in the multivariable Cox regression model.

Differences were considered significant at *P* < 0.05. Statistical analyses were performed using SAS software, Version 9.4 of the SAS System (SAS Institute, Cary, NC, USA).

## Supplementary Information

Supplementary Information.

## Data Availability

All data containing relevant information to support the study findings are provided in the manuscript.
