# Supplementary material for: Sex stratification of the trends and risk of mortality among individuals living with HIV under different transmission categories
Source: Sci Rep. 2022 Jun 3;12:9266. doi: 10.1038/s41598-022-13294-y (PMC9166722; doi:10.1038/s41598-022-13294-y)
Supplement: Supplementary file 1 — Supplementary Information. [file 41598_2022_13294_MOESM1_ESM.docx]

Supplementary Table S1. Association of HIV transmission category (heterosexuals and PWID) with all-cause mortality in different multivariable models.

|  | Model 1^a^ | | Model 2^b^ | | Model 3^c^ | | Model 4^d^ | |
| --- | --- | --- | --- | --- | --- | --- | --- | --- |
|  | aHR (95% CI) | *P*-Value | aHR (95% CI) | *P*-Value | aHR (95% CI) | *P*-Value | aHR (95% CI) | *P*-Value |
| PWID | Reference |  | Reference |  | Reference |  | Reference |  |
| Heterosexuals | 0.35  (0.32-0.39) | <0.001 | 0.52  (0.47-0.58) | <0.001 | 0.92  (0.83-1.02) | 0.098 | 1.17  (1.06-1.29) | 0.002 |

^a^Model 1 include age group, sex, period of HIV diagnosis, HIV transmission category, marital status, occupation, specimen source, HIV diagnosis region, and AIDS event.

^b^Model 2 include sex, period of HIV diagnosis, HIV transmission category, marital status, occupation, specimen source, HIV diagnosis region, and AIDS event.

^c^Model 3 include age group, sex, period of HIV diagnosis, HIV transmission category, marital status, occupation, specimen source, and HIV diagnosis region.

^d^Model 4 include sex, period of HIV diagnosis, HIV transmission category, marital status, occupation, specimen source, and HIV diagnosis region.

Abbreviation:

aHR, adjusted hazard ratio; CI, confidence interval; PWID, people who inject drugs.

In model 4 (excluding age group and AIDS event), the aHR of heterosexual (vs. PWID) on the all-cause mortality (1.17, 95% CI 1.06-1.29) is similar to the crude HR in all set (1.16, 95% CI 1.08-1.23) (Table 2). The aHR of heterosexual (vs. PWID) on the all-cause mortality was reversed in model 3 (adding age group) (0.92, 95% CI 0.83-1.02), model 2 (adding AIDS event) (0.52, 95% CI 0.47-0.58), and model 3 (adding both age group and AIDS event) (0.35, 95% CI 0.32-0.39).
